# Design of Fluxgate Current Sensor Based on Magnetization Residence Times and Neural Networks

**DOI:** 10.3390/s24123752

**Published:** 2024-06-09

**Authors:** Jingjie Li, Wei Ren, Yanshou Luo, Xutong Zhang, Xinpeng Liu, Xue Zhang

**Affiliations:** 1 Key Laboratory of Intelligent Control and Optimization for Industrial Equipment of Ministry of Education, Dalian University of Technology, Dalian 116024, China; lijingjie@dlut.edu.cn (J.L.); tm423@mail.dlut.edu.cn (X.Z.); 3232339879@mail.dlut.edu.cn (X.L.); 2 School of Control Science and Engineering, Dalian University of Technology, Dalian 116024, China; 3 Beijing Institute of Aerospace Systems Engineering, Beijing 100076, China

**Keywords:** current sensor, fluxgate, residence times, neural network, linearity error

## Abstract

This study introduces a novel fluxgate current sensor with a compact, ring-shaped configuration that exhibits improved performance through the integration of magnetization residence times and neural networks. The sensor distinguishes itself with a unique magnetization profile, denoted as M waves, which emerge from the interaction between the target signal and ambient magnetic interference, effectively enhancing interference suppression. These M waves highlight the non-linear coupling between the magnetic field and magnetization residence times. Detection of these residence times is accomplished using full-wave rectification circuits and a Schmitt trigger, with a digital output provided by timing sequence detection. A dual-layer feedforward neural network deciphers the target signal, exploiting this non-linear relationship. The sensor achieves a linearity error of 0.054% within a measurement range of 15 A. When juxtaposed with conventional sensors utilizing the residence-time difference strategy, our sensor reduces linearity error by more than 40-fold and extends the effective measurement range by 150%. Furthermore, it demonstrates a significant decrease in ambient magnetic interference.

## 1. Introduction

Current detection is critical to the smooth and secure operation of various electrical and electronic systems [1,2,3,4,5]. A variety of current sensors, grounded in principles such as Ohm’s law and Faraday’s law of induction, play a pivotal role in accurately measuring current flow in circuits. These include shunt resistors [6], current transformers [7], Rogowski coils [3], Hall effect sensors [8], fluxgate sensors [4], magnetoresistive sensors [9], and fiber optic sensors [10]. Among them, fluxgate current sensors, recognized for their superior sensitivity and minimal thermal drift, are extensively employed across various industries, including automotive, power electronics, smart grids, and industrial automation [4].

Currently, electric vehicles (EVs) are gaining increasing popularity in the automotive landscape. These vehicles rely heavily on precise and accurate current measurements for essential functions such as battery management, motor control, autonomous driving, and charging systems [11,12,13,14,15]. Fluxgate current sensors, known for their high precision, wide bandwidth (including DC), and galvanic isolation, are particularly favored in this context [16,17,18]. These sensors have become a focal point in research aimed at enhancing performance. An innovative approach involves using a 20 µm-thick ${\mathrm{Fe}}_{15}$${\mathrm{Ni}}_{46}$${\mathrm{Co}}_{39}$ film with low coercivity and high Curie temperature, placed on a Cu ring to serve as a magnetic core [19]. Garcha P. developed a scalable integrated fluxgate current sensor employing a mixed-signal architecture for improved energy efficiency [20]. In [21], a micro-fluxgate and a current transformer were combined to extend the bandwidth of current measurement. A specified periodic current was injected into the output port of the current sensor to cancel the magnetization distortion [22]. The bandwidth and zero-point stability of the closed-loop residual current sensor were enhanced by increasing the voltage of the AC winding and optimizing the demodulation circuit [23]. By integrating these approaches and advancements, researchers aim to develop more accurate, stable, and responsive sensors, catering to the increasingly demanding needs of modern technological applications.

Fluxgate current sensors, which are variations of magnetic field sensors, are susceptible to interference from both geomagnetic and power frequency magnetic fields, posing challenges, particularly in measuring weak currents [24,25,26,27]. This interference can jeopardize safety by causing erratic motor behavior and potential vehicle breakdowns, which is especially concerning in autonomous driving scenarios. To mitigate these effects, strategies such as sensor array schemes have been explored and experimentally validated [25,26,28,29]. Additional research has been directed towards innovating fluxgate magnetic cores by integrating structures for field shielding or flux guidance, exemplified by designs like U-shaped, split, and double cores [24,27,30,31]. While many previous studies have effectively mitigated magnetic field interference, the challenge of eliminating such interference from a mechanistic perspective and achieving a high-performance, low-cost sensor design remains a significant issue in applications.

The residence times difference (RTD) fluxgate evaluates weak magnetic fields by monitoring the residence time within the metastable attractors of a magnetization system in the presence of a time-periodic excitation signal [32,33]. Through ongoing research in sensing mechanisms [34,35], modeling analysis [36], detection methods [37], topology [38], data processing [39], and other areas, RTD technology has gradually matured. It is increasingly replacing traditional harmonic fluxgate in applications requiring miniaturized and low-cost sensing devices, such as biomedical and industrial non-destructive testing [40,41,42]. The development of RTD fluxgate creates favorable conditions for performance optimization of the current sensors.

In this paper, we introduce a novel fluxgate current sensor featuring a compact ring-shaped design, which employs a magnetization residence times readout strategy enhanced by neural networks. Theoretical modeling demonstrates that this sensor can inherently measure both the target current and external magnetic fields using distinct features. Consequently, it utilizes this coupled information to effectively mitigate interference from external sources. Due to the complex nonlinearity between the target current and external magnetic fields, a neural network is employed to distinguish the current characteristics from the coupled data, thereby significantly improving detection accuracy and reducing interference, as confirmed by experimental validation. Moreover, the designed sensor facilitates a purely digital output using minimal circuitry, eliminating the need for an AD converter. In conclusion, the main contributions of this paper are as follows:

1. We have observed and comprehensively explained the unique ‘M’-shaped magnetization within the fluxgate current sensor for the first time, employing our proposed magnetic microelements method.
2. We have established theoretical support for current sensing and ambient interference suppression by analyzing the nonlinear coupling between magnetization residence times and the magnetic field within the M waveforms.
3. We have integrated neural networks with the sensing mechanism innovatively to facilitate high-precision target current extraction.

This article is structured as follows. Section 2 outlines the basic architecture of the sensor probe. Section 3 presents a comprehensive theoretical analysis and detection strategy, with a detailed exploration of magnetization residence times. Section 4 delves into circuit design, encompassing current driving, conditioning, and time-sequence detection. Section 5 presents the experimental setup and data analysis methodologies. Section 6 provides the conclusion and discusses future research directions.

## 2. Structure of the Sensor Probe

**Figure 1:**
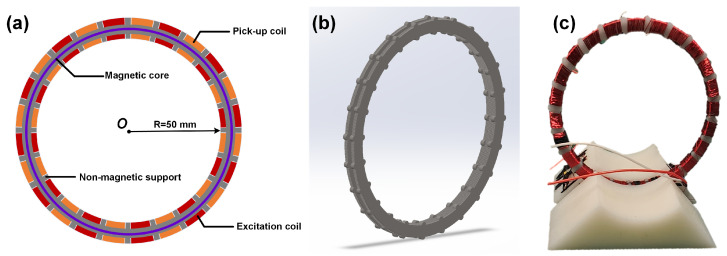
The structure of the proposed current sensor probe. (**a**) The structure of the current sensor. (**b**) The ring-shaped support skeleton. (**c**) The sensor probe prototype.

The sensor probe, typically elongated for fluxgate utilizing RTD readout stragety [33,35], has been redesigned into a circular form to better align with the magnetic field pattern generated by an elongated, current-carrying straight wire. This reconfiguration is illustrated in Figure 1a, where the novel fluxgate current sensor consists of a nonmagnetic support skeleton, a magnetic core, an excitation coil, and a pick-up coil. The magnetic core, composed of a 20 µm-thick cobalt-based amorphous alloy with the composition ${\mathrm{Co}}_{65}$${\mathrm{Fe}}_{5}$${\mathrm{Cr}}_{2}$${\mathrm{Si}}_{18}$$B_{10}$, is annealed in a longitudinal magnetic field to increase dynamic permeability and reduce coercivity. The saturation magnetic field density and coercivity of the self-made magnetic material are approximately 0.48 T and 0.22 A/m, respectively, and the maximum permeability exceeds 0.58 million. Parameters of the magnetic materials used in the magnetic core are summarized in Table 1. The ring-shaped support alternates sections to accommodate the 480-turn excitation and pick-up coils. This support is divided into 24 equal segments as depicted in Figure 1b, and the completed sensor probe assembly is shown in Figure 1c.

**Figure 2:**
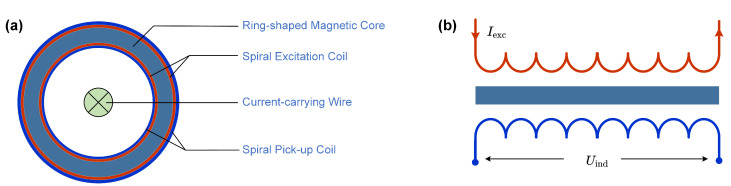
The equivalent model of the fluxgate current sensor. (**a**) The equivalent structure. (**b**) The equivalent model.

As shown in Figure 2, the current-carrying wire is placed at the center of the ring to transmit the target current. During operation, a sinusoidal excitation current ($I_{\mathrm{exc}}$) is fed into the excitation coil, creating a periodic magnetic field. This causes the magnetic core to continuously switch between positive and negative magnetic saturation states. Consequently, the fluctuating magnetic flux in the pick-up coil induces a voltage output ($U_{\mathrm{ind}}$).

## 3. Theoretical Support and Detection Strategy

### 3.1. Theoretical Modeling

According to the Biot–Savart law, the magnetic field $H_{\mathrm{dut}}$ generated by an elongated, straight wire carrying a current $I_{\mathrm{dut}}$ is expressed as follows:

(1) $$H_{\mathrm{dut}}=\gamma I_{\mathrm{dut}},$$

where γ=1/(2πr), $I_{\mathrm{dut}}$ is the target current to be measured, *r* is the distance to the wire center.

The excitation coil, modeled as a solenoid with a turn density of $n_{\mathrm{exc}}$, is designed with a magnetic core at its center. Upon introducing a sinusoidal current, the coil generates an excitation magnetic field, $H_{\mathrm{exc}}$, which is defined as follows:

(2) $$H_{\mathrm{exc}}=n_{\mathrm{exc}}I_{\mathrm{exc}}=n_{\mathrm{exc}}A_{\mathrm{exc}}\sin\left(2\pi f_{\mathrm{exc}}t\right)=\left|H_{\mathrm{exc}}\right|\sin\left(2\pi f_{\mathrm{exc}}t\right),$$

where $I_{\mathrm{exc}}=A_{\mathrm{exc}}\sin\left(2\pi f_{\mathrm{exc}}t\right)$ is the excitation current, and $A_{\mathrm{exc}},\;f_{\mathrm{exc}}$ are respectively the amplitude and frequency.

**Figure 3:**
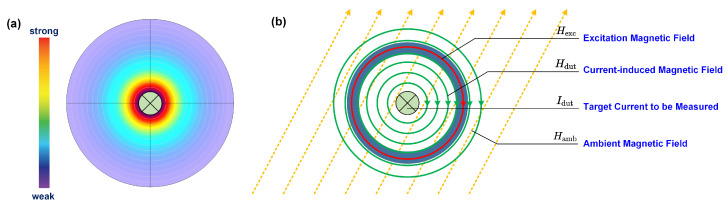
The magnetic fields involved in the current sensor. (**a**) The magnetic field generated by the target current $I_{\mathrm{dut}}$ (by COMSOL Multiphysics 6.2). (**b**) The distribution of magnetic fields.

Figure 3 illustrates the presence of three magnetic fields surrounding the magnetic core: the excitation magnetic field $H_{\mathrm{exc}}$, the magnetic field induced by the current $H_{\mathrm{dut}}$, and the ambient magnetic field $H_{\mathrm{amb}}$. The ambient magnetic field is represented by dashed orange lines, the excitation magnetic field by a solid red line within the toroidal magnetic core, and the magnetic field induced by the current by solid green lines in a concentric arrangement with the excitation field.

**Figure 4:**
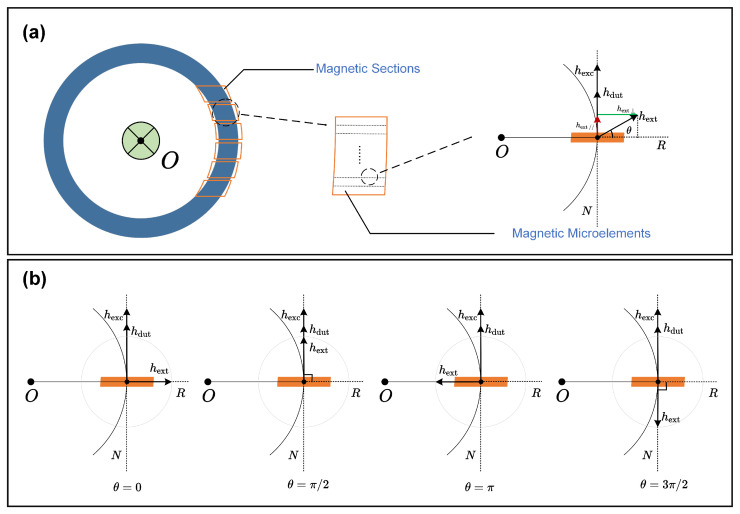
The depiction of the magnetic segments and microelements approach. (**a**) The magnetic sections and microelements. (**b**) The magnetic microelements with different angles.

To analyze the distribution of magnetic induction, the magnetic core is divided into several sections, each comprising numerous microelements, as illustrated in Figure 4a. Each microelement is considered a homogeneous magnetic point, maintaining constant orientation for both the excitation and observed fields perpendicular to the magnetic core’s normal direction *N* at that point. The orientation of the ambient magnetic field $H_{\mathrm{amb}}$ relative to the radial direction *R* at any given point is denoted by θ, which varies from 0 to 2π as shown in Figure 4b. The total magnetic field H(θ) at each magnetic point is calculated as follows:

(3) $$H\left(\theta \right)=H_{\mathrm{dut}}+H_{\mathrm{exc}}+H_{\mathrm{amb}}\sin\theta .$$

The magnetic core is fabricated from a cobalt-based alloy film, a soft magnetic material characterized by high magnetic permeability and low coercivity. The arc tangent model, based on the shape characteristics of ferromagnetic hysteresis, is developed using trigonometric functions [36,43,44], as depicted in the following equation:

(4) $$B\left(H\right)=\alpha \arctan\left[\beta \left(H\pm H_{c}\right)\right],$$

where α is the saturation flux density, β is the magnetic permeability, and $H_{c}$ is the magnetic coercive force. Integrating Equations (3) and (4), the magnetic induction B(θ) for each magnetic microelement is expressed by the following equation:

(5) $$\begin{array}{c} \phi \left(\theta \right)=B\left(H\left(\theta \right)\right)S=\alpha \arctan\left[\beta \left(H_{\mathrm{dut}}+H_{\mathrm{exc}}+H_{\mathrm{amb}}\sin\theta \pm H_{c}\right)\right]S, \end{array}$$

where *S* is the cross-section area of the pick-up coil.

As illustrated in Figure 4a, the total magnetic flux of the pick-up coil is calculated by integrating Equation (5) from θ=0 to 2π,

(6) $$\begin{array}{cc} \Phi \left(t\right)= & N\int_{0}^{2\pi }\phi \left(\theta \right)d\theta =N\int_{0}^{2\pi }\alpha \arctan\left[\beta \left(H_{\mathrm{dut}}+H_{\mathrm{exc}}+H_{\mathrm{amb}}\sin\theta \pm H_{c}\right)\right]Sd\theta , \end{array}$$

where *N* is the winding turns of the pick-up coil.

### 3.2. Numerical Analysis

In accordance with Faraday’s law of induction and Equation (6), the voltage output of the pick-up coil can be theoretically derived. However, the non-linear nature of Equation (6) makes it too complicated to obtain a precise analytical solution for the induced output. In this context, we assume that the magnetic fields induced by the current, $H_{\mathrm{ind}}$, and the ambient magnetic field, $H_{\mathrm{amb}}$, behave as DC signals. Drawing on Equation (5), the voltage output e(t,θ) for each magnetic microelement is articulated as follows:

(7) $$\begin{array}{c} e\left(t,\theta \right)=-N\frac{d\phi (\theta )}{dt}=-NS\left(\frac{dB}{dH}\frac{dH_{\mathrm{exc}}}{dt}+\left((H_{\mathrm{dut}}+H_{\mathrm{exc}}+H_{\mathrm{amb}}\right)\frac{d^{2}B}{dH^{2}}\frac{dH_{\mathrm{exc}}}{dt}\right). \end{array}$$

The voltage $e\left(t\right)$ yield from the pick-up coil can be determined by integrating Equation (7) from θ=0 to θ=2π,

(8) $$\begin{array}{c} e\left(t\right)=\int_{0}^{2\pi }e\left(t,\theta \right)d\theta =NS\int_{0}^{2\pi }\frac{dB\left(\theta \right)}{dt}d\theta \approx NS\sum_{i=1}^{n}\frac{dB\left(\theta \right)}{dt}, \end{array}$$

where *n* is the number of the microelements.

**Figure 5:**
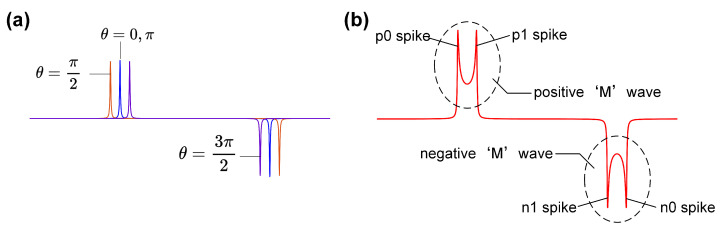
The numerical analysis of the sensor output. (**a**) The output of the selected magnetic microelement, θ=0,π/2,π,3π/2. (**b**) The M waves in the sensor output.

Utilizing Equation (8) and employing numerical differentiation and integration, we determine the sensor voltage output, as illustrated in Figure 5. As shown in Figure 5a, each magnetic microelement generates a pair of positive and negative spikes within one excitation cycle. The spikes p0 and n0, originating from the same magnetic microelement, constitute a spike pair, similar to the spikes p1 and n1. Figure 5b reveals that the coil output encompasses a positive and a negative M wave per cycle, with the positive M wave featuring spikes p0 and p1, and the negative M wave showcasing spikes n0 and n1.

The induced output displays positive spikes upon reaching $+H_{c}$ and negative spikes at $-H_{c}$, as defined by the characteristics of magnetic hysteresis detailed in Equation (5). Using Equation (3), the conditions for positive spikes in each magnetic microelement are specified as follows:

(9) $$\begin{array}{c} H_{\mathrm{dut}}+H_{\mathrm{exc}}+H_{\mathrm{amb}}\sin\theta =+H_{c},\;\frac{\partial H_{\mathrm{exc}}}{\partial t}>0, \end{array}$$

while the negative spikes appear at

(10) $$\begin{array}{c} H_{\mathrm{dut}}+H_{\mathrm{exc}}+H_{\mathrm{amb}}\sin\theta =-H_{c},\;\frac{\partial H_{\mathrm{exc}}}{\partial t}<0. \end{array}$$

By integrating Equations (2), (9) and (10), the times for positive ($t_{p}$) and negative ($t_{n}$) spikes are represented as follows:

(11) $$\left\{\begin{array}{cc} t_{p} & =\frac{1}{2\pi f_{\mathrm{exc}}}\arcsin\left(\frac{H_{c}-H_{\mathrm{amb}}\sin\theta -H_{\mathrm{dut}}}{\left|H_{\mathrm{exc}}\right|}\right),\\ t_{n} & =\frac{1}{2\pi f_{\mathrm{exc}}}\arcsin\left(\frac{H_{c}+H_{\mathrm{amb}}\sin\theta +H_{\mathrm{dut}}}{\left|H_{\mathrm{exc}}\right|}\right)+\frac{1}{2f_{\mathrm{exc}}}. \end{array}\right.$$

The primary and secondary derivatives of *B* with respect to *H* are obtained from Equation (4),

(12) $$\frac{dB}{dH}=\frac{\alpha \beta }{\beta^{2}{\left(H\pm H_{c}\right)}^{2}+1},\;\frac{d^{2}B}{dH^{2}}=\frac{-2\alpha \beta^{3}\left(H\pm H_{c}\right)}{{\left(\beta^{2}{\left(H\pm H_{c}\right)}^{2}+1\right)}^{2}}.$$

The following relationship is derived from Equation (12):

(13) $${\left.\frac{dB}{dH}\right|}_{H=\pm H_{c}}=\mu_{\max},\;{\left.\frac{d^{2}B}{dH^{2}}\right|}_{H=\pm H_{c}}=0.$$

Considering Equations (2), (7), (9), (10) and (13), the spike peak-voltage $e_{s}\left(\theta \right)$ for each magnetic microelement can be determined as follows:

(14) $$\begin{array}{c} e_{s}\left(\theta \right)=-2\pi NS\left|H_{\mathrm{exc}}\right|f_{\mathrm{exc}}\mu_{\max}\cos\left(2\pi f_{\mathrm{exc}}t_{s}\right), \end{array}$$

where $t_{s}$ is either $t_{p}$ or $t_{n}$.

**Figure 6:**
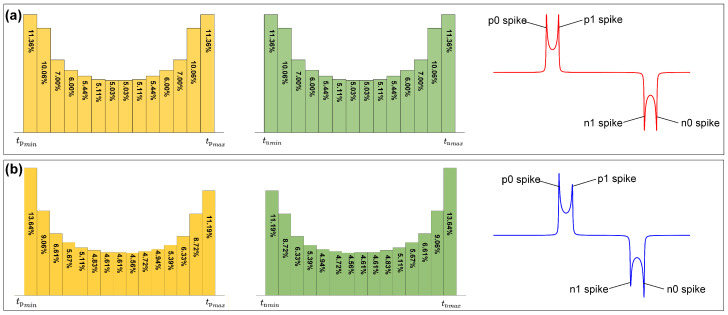
The sensor output and the distribution of spike times. (**a**) The sensor output when $H_{\mathrm{dut}}=0$. (**b**) The sensor output when $H_{\mathrm{dut}}\neq 0$.

In line with Equation (11), the times $t_{p}$ and $t_{n}$ vary along with θ. Under normal operating conditions, where $\left|H_{\mathrm{exc}}\right|\gg H_{\mathrm{dut}}$ and $\left|H_{\mathrm{exc}}\right|\gg H_{\mathrm{amb}}$, $t_{p}$ and $t_{n}$ are approximated as constants across the range of θ from 0 to 2π within an excitation cycle. Together with Equation (14), the peak value for each magnetic microelement is considered constant. Based on this approximation, the sensor output is predominantly influenced by the distribution of spike times. The distribution probability of spike times for each microelement, as well as the numerical representation of the voltage of the pickup coil amid varying magnetic fields $H_{\mathrm{dut}}$, is presented in Figure 6.

As shown in Figure 6, the output waveform mirrors the distribution of spike times. For $H_{\mathrm{dut}}=0$, the M waves display symmetric spikes. However, if $H_{\mathrm{dut}}\neq 0$, then an asymmetry emerges between the spikes of p0 and p1, and a similar difference can be observed between the spikes of n0 and n1.

### 3.3. Residence Times of Magnetization States

The magnetization of each microelement alternates regularly between positive and negative saturation, inducing spike-like responses in the pick-up coils. As indicated by Equation (11), the time locations of the spikes are influenced by both the detected magnetic field $H_{\mathrm{dut}}$ and the ambient magnetic field $H_{\mathrm{amb}}$. Within this framework, the duration spent in various magnetic states serves as a means to measure the target field.

**Figure 7:**
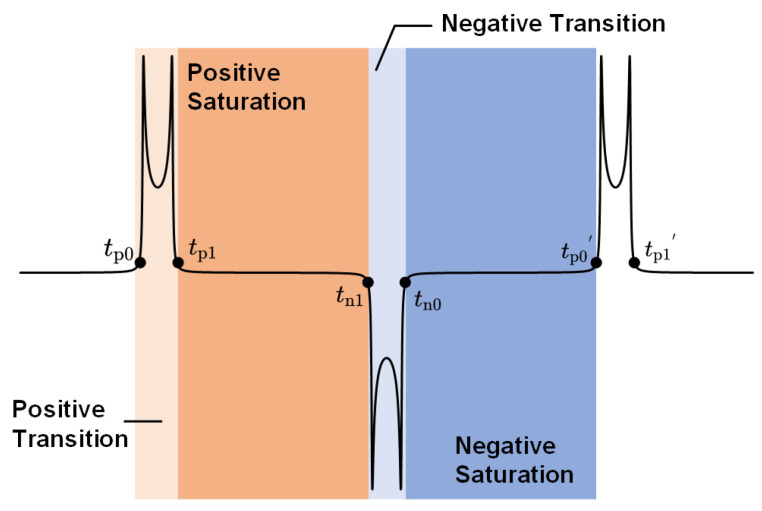
The magnetization states and the locations of the feature spikes in sensor output.

As depicted in Figure 7, throughout an excitation cycle, the magnetic core undergoes four states: positive transition, positive saturation, negative transition, and negative saturation. The times $t_{p0}$, $t_{p1}$, $t_{n1}$, and $t_{n0}$ correspond to the occurrences of the spikes p0, p1, n1, and n0, respectively. Based on Equation (11), the following can be derived:

(15) $$\left\{\begin{array}{cc} t_{p0} & =\min\left(t_{p}\right)=\frac{1}{2\pi f_{\mathrm{exc}}}\arcsin\left(H_{1}\right),\\ t_{p1} & =\max\left(t_{p}\right)=\frac{1}{2\pi f_{\mathrm{exc}}}\arcsin\left(H_{2}\right),\\ t_{n0} & =\max\left(t_{n}\right)=\frac{1}{2\pi f_{\mathrm{exc}}}\arcsin\left(H_{4}\right)+\frac{1}{2f_{\mathrm{exc}}},\\ t_{n1} & =\min\left(t_{n}\right)=\frac{1}{2\pi f_{\mathrm{exc}}}\arcsin\left(H_{3}\right)+\frac{1}{2f_{\mathrm{exc}}},\\ t_{p0}^{\prime } & =t_{p0}+\frac{1}{f_{\mathrm{exc}}}=\frac{1}{2\pi f_{\mathrm{exc}}}\arcsin\left(H_{1}\right)+\frac{1}{f_{\mathrm{exc}}},\\ t_{p1}^{\prime } & =t_{p1}+\frac{1}{f_{\mathrm{exc}}}=\frac{1}{2\pi f_{\mathrm{exc}}}\arcsin\left(H_{2}\right)+\frac{1}{f_{\mathrm{exc}}}. \end{array}\right.$$

where $H_{1}:=\left(H_{c}-H_{\mathrm{amb}}-H_{\mathrm{dut}}\right)/\left|H_{\mathrm{exc}}\right|$, $H_{2}:=\left(H_{c}+H_{\mathrm{amb}}-H_{\mathrm{dut}}\right)/\left|H_{\mathrm{exc}}\right|$, $H_{3}:=\left(H_{c}-H_{\mathrm{amb}}+H_{\mathrm{dut}}\right)/\left|H_{\mathrm{exc}}\right|$ and $H_{4}:=\left(H_{c}+H_{\mathrm{amb}}+H_{\mathrm{dut}}\right)/\left|H_{\mathrm{exc}}\right|$. Combining Equation (15) with Figure 7, the residence time of each magnetization state can be given as follows:

(16) $$\left\{\begin{array}{cc} t_{\mathrm{PT}} & =t_{p1}-t_{p0}=\frac{1}{2\pi f_{\mathrm{exc}}}\left(\arcsin\left(H_{2}\right)-\arcsin\left(H_{1}\right)\right),\\ t_{\mathrm{PS}} & =t_{n1}-t_{p1}=\frac{1}{2\pi f_{\mathrm{exc}}}\left(\arcsin\left(H_{3}\right)-\arcsin\left(H_{2}\right)\right)+\frac{1}{2f_{\mathrm{exc}}},\\ t_{\mathrm{NT}} & =t_{n0}-t_{n1}=\frac{1}{2\pi f_{\mathrm{exc}}}\left(\arcsin\left(H_{4}\right)-\arcsin\left(H_{3}\right)\right),\\ t_{\mathrm{NS}} & =t_{p0}^{\prime }-t_{n0}=\frac{1}{2\pi f_{\mathrm{exc}}}\left(\arcsin\left(H_{1}\right)-\arcsin\left(H_{4}\right)\right)+\frac{1}{2f_{\mathrm{exc}}}, \end{array}\right.$$

where $t_{\mathrm{PT}}$, $t_{\mathrm{PS}}$, $t_{\mathrm{NT}}$, and $t_{\mathrm{NS}}$ are the duration of the positive transition, the positive saturation, the negative transition, and the negative saturation, respectively.

The residence times difference (RTD) [33] between the positive region and negative region can be expressed as follows:

(17) $$\begin{array}{cc} RTD & =\left(t_{\mathrm{PT}}+t_{\mathrm{PS}}\right)-\left(t_{\mathrm{NT}}+t_{\mathrm{NS}}\right)=\frac{1}{\pi f_{\mathrm{exc}}}\left(\arcsin\left(H_{3}\right)-\arcsin\left(H_{1}\right)\right). \end{array}$$

Based on the Taylor expansion, in the vicinity of zero, the linear approximation arcsin(x)≈x is applicable. Consequently, when $H_{c}+\left|H_{\mathrm{amb}}\right|+\left|H_{\mathrm{dut}}\right|\ll \left|H_{\mathrm{exc}}\right|$, Equation (17) can be approximately reformulated as follows:

(18) $$\begin{array}{c} RTD\approx \frac{2H_{\mathrm{dut}}}{\pi f_{\mathrm{exc}}\left|H_{\mathrm{exc}}\right|}. \end{array}$$

Subsequently, by combining Equation (1), the current detection sensitivity is determined as follows:

(19) $$\begin{array}{c} \frac{\partial RTD}{\partial I_{\mathrm{dut}}}\approx \frac{2\gamma }{\pi f_{\mathrm{exc}}\left|H_{\mathrm{exc}}\right|}. \end{array}$$

The relationship between the difference in residence times and the target current in the near-zero range is linear, as indicated by Equation (19).

### 3.4. Neural Networks-Based Detection

**Figure 8:**
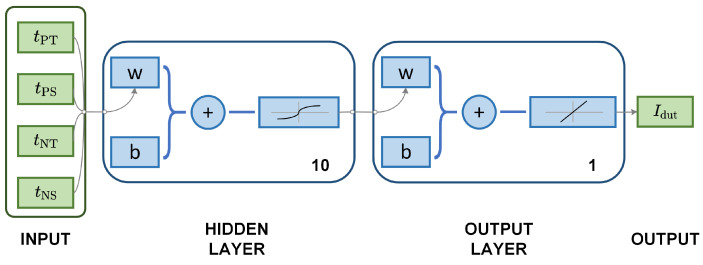
The neural networks for the target current detection.

The detection of the induced field $H_{\mathrm{dut}}$ in our proposed sensor, as detailed in Equation (16), presents a multi-input nonlinear challenge. Both the magnetic coercive force $H_{c}$ and the ambient magnetic field $H_{\mathrm{amb}}$ influence the residence times. Notably, $H_{c}$ is considered constant for a given magnetic material. Hence, the residence time attributes are leveraged to assess $H_{\mathrm{amb}}$ and mitigate ambient magnetic field interference, a feature termed “inherent suppression of ambient magnetic field interference”. Figure 8 demonstrates the use of neural networks to ascertain the target current $I_{\mathrm{dut}}$ from residence times. The designed neural network is a two-layer forward propagation model, consisting of ten hidden layers of sigmoid neurons and linear output neurons, trained using the Bayesian regularization method.

## 4. Circuits Design

### 4.1. Current Drive Circuit for Excitation Coil

**Figure 9:**
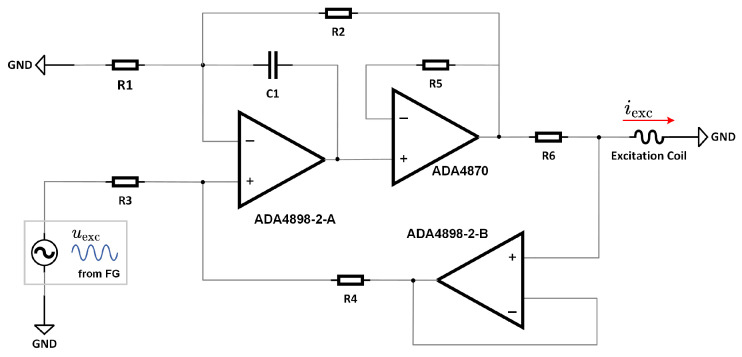
The composite amplifier-enhanced Howland current source.

For the proposed current sensor to function stably, its magnetic core must periodically alternate between positive and negative saturation states. Consequently, a periodic current signal must be supplied to the excitation coil. To generate a precise excitation current, a Composite Amplifier-Enhanced Howland Current Source (CAEHCS) is utilized, aiming to diminish the output offset current and bias current. Figure 9 demonstrates that the CAEHCS, leveraging the synergy of the low-noise amplifier ADA4898-2 and the high-current output driver ADA4870 from Analog Devices, Massachusetts, USA, can produce a current output of up to 1 A. It also achieves an output error as low as 0.2 mA and maintains a constant bandwidth of 8 MHz, assuming that the gain resistance is configured as $R_{1}/R_{2}=R_{3}/R_{4}=k,R_{1}//R_{2}=R_{3}//R_{4}$. The output current supplied to the excitation coil is defined by $i_{\mathrm{exc}}=u_{\mathrm{exc}}/\left(kR_{6}\right)$.

### 4.2. Conditioning Circuit for Residence Times Detection

**Figure 10:**
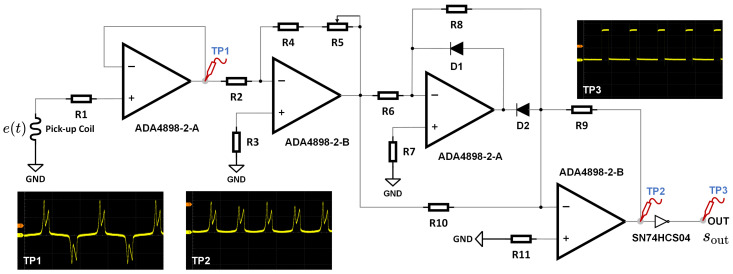
The signal conditioning circuit based on precision full-wave rectification.

Building on Equation (15) and the proposed neural network-based detection approach, designing a signal conditioning circuit capable of extracting the magnetization residence times from the analog output of the pick-up coil e(t) is essential. We introduce a streamlined yet effective circuit layout that obviates the need for an analog-to-digital converter (ADC). The circuit comprises two key sections: the shaping and the triggering components. As depicted in Figure 10, the shaping section consists of a voltage follower, an inverting amplifier, a precision full-wave rectification module, and an inverter equipped with a Schmitt trigger input. This configuration effectively converts the pick-up coil’s output into a rectangular wave $s_{\mathrm{out}}$, facilitating the determination of magnetization residence times.

### 4.3. Timing Sequence Detection

**Figure 11:**
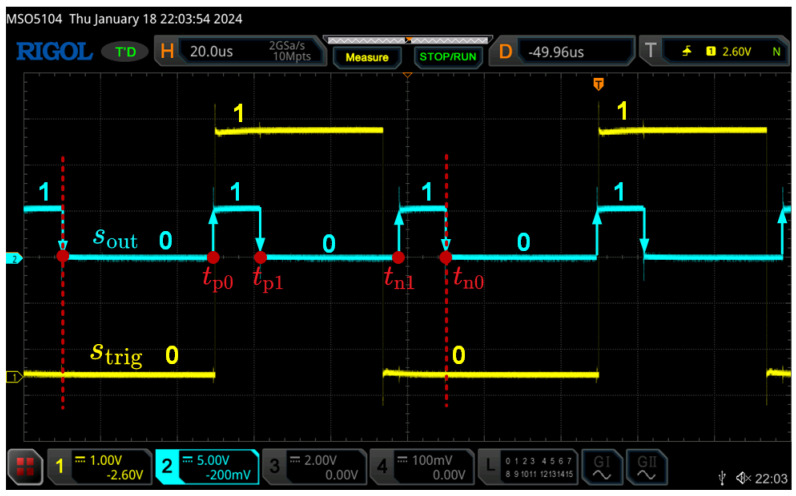
The timing sequence of the rectangular output and the trigger signal.

To identify the onset of the rectangular wave and ascertain the target current’s polarity, a NOT gate is utilized to reshape the excitation voltage and generate the trigger signal $s_{\mathrm{trig}}$. Figure 11 demonstrates that $t_{n0}$ is clearly recognizable within a single excitation cycle when $s_{\mathrm{out}}$ experiences a falling edge and $s_{\mathrm{trig}}$ is at zero. Following this, the timings of all spikes can be recorded, and as outlined in Equation (16), the duration of each magnetic state can be determined. These processes can be efficiently implemented using digital I/Os.

## 5. Results

### 5.1. Experimental Setup

**Figure 12:**
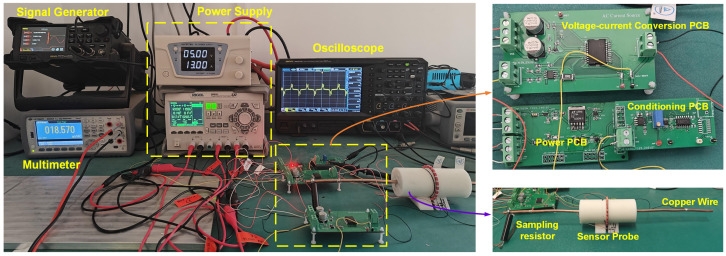
The experimental arrangement comprises the experimental instruments, sensor probe, and detection circuitry.

The experimental setup is illustrated in Figure 12. A printed circuit board (PCB) designed for voltage-current transformation processes the sinusoidal voltage output from the DG2102 signal generator(product from Rigol, China), converting it to the necessary current signal that powers the excitation coil. Throughout the experiment, the excitation current’s frequency and peak-to-peak amplitude were maintained at 10 kHz and 200 mA, respectively. A $6_{1/2}$ digit multimeter, model 34460A (product from Keysight, Santa Rosa, CA, USA), equipped with a low temperature drift sampling resistor, was used to determine the precise value of the target current. For circuit troubleshooting and data capture, we used the MS05104 digital oscilloscope (product from Rigol, Beijing, China). Two power supplies, the DP832 (product from Rigol, Beijing, China) and the KPS1560D (product from Wanptek, Shenzhen, China), operating in constant current (CC) mode, provided the calibration target current. Calibration was carried out using a straight copper wire, which carried a safe threshold current of up to 20 A and measured 5.0 mm in radius.

### 5.2. Experimental Data and Traditional RTD Estimation

**Figure 13:**
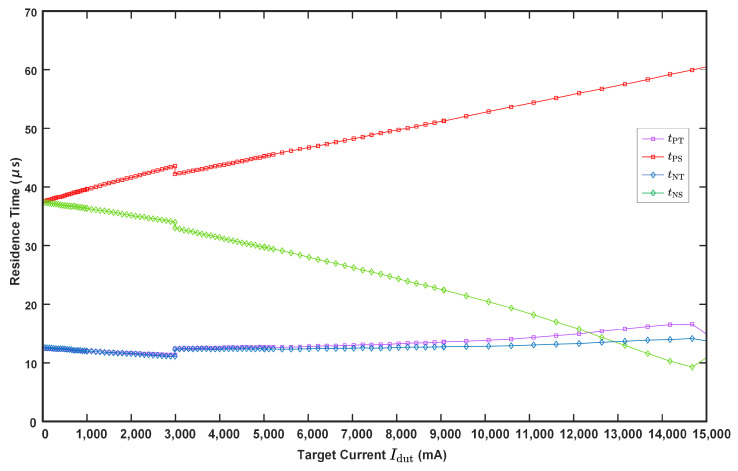
The experimental data.

Adjusting the target current allows us to elicit variable responses from the sensor, as depicted in Figure 13. The data illustrate that the sensor possesses a measurement range up to 15 A. Variations in the target current result in more pronounced changes in the residence time of the magnetic saturation states than those of the transition states. Notably, data anomalies are observed near $I_{\mathrm{dut}}=3000$ mA. At this point, the four magnetization residence times $t_{\mathrm{PS}}$, $t_{\mathrm{PT}}$, $t_{\mathrm{NS}}$ and $t_{\mathrm{NT}}$ change simultaneously, with $t_{\mathrm{PT}}$ and $t_{\mathrm{NT}}$ exhibiting significant changes than usual. And Equation (16) indicates that $t_{\mathrm{PT}}$ and $t_{\mathrm{PT}}$ are more sensitive to variations in the ambient magnetic field than to changes in the target current magnetic field. Thus, the observed discontinuity is mainly attributed to changes in the ambient magnetic field. These perturbations are inevitable, stemming from both the omnipresent geomagnetic field and its temporal fluctuations.

**Figure 14:**
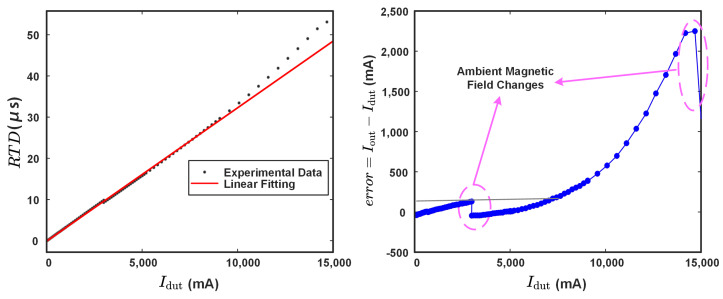
The sensor characteristics. (**a**) The sensor calibration curve, (**b**) The sensor output error.

The target current is quantified by employing the RTD approximation outlined in Equation (19). Linear regression facilitates the establishment of a relationship between RTD and the target current. Figure 14 displays the calibration results, including output behavior and adjustment error, which suggest a sensitivity correlation of approximately $I_{\mathrm{dut}}=319.08\times RTD-16.63$. Within a 6 A measurement range, the correlation demonstrates high linearity, with the calibration error remaining below 2.17%. However, as the target current increases beyond this range, the sensor exhibits a pronounced nonlinearity, rendering it unsuitable for industrial applications. As depicted in Figure 14b, the disruptive influence of ambient magnetic field variations is significant during linear regression, leading to substantial measurement errors, particularly in weak current detection.

### 5.3. Neural Networks Training and Sensor Calibration

**Figure 15:**
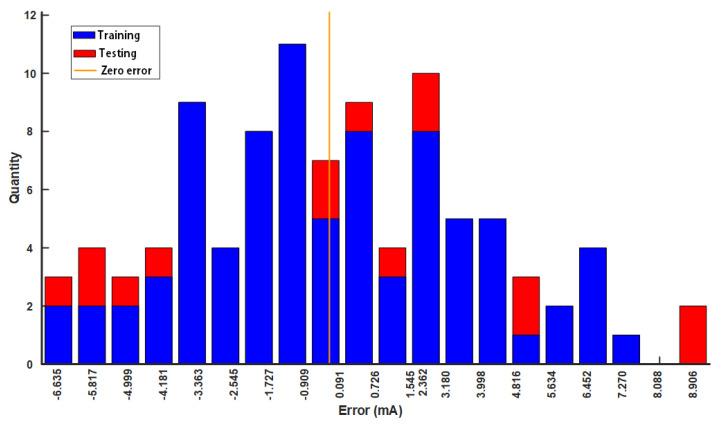
The histogram of training error and testing error in the neural networks.

The proposed neural network, depicted in Figure 8, is trained using the experimental data from Figure 13, leading to the formulation of the relationship $I_{\mathrm{out}}=\mathrm{NNF}\left(t_{\mathrm{PT}},t_{\mathrm{PS}},t_{\mathrm{NT}},t_{\mathrm{NS}}\right)$. Figure 15 presents a histogram that depicts the distribution of training and testing errors, demonstrating that the error margin of the neural network is limited to ±10 mA.

**Figure 16:**
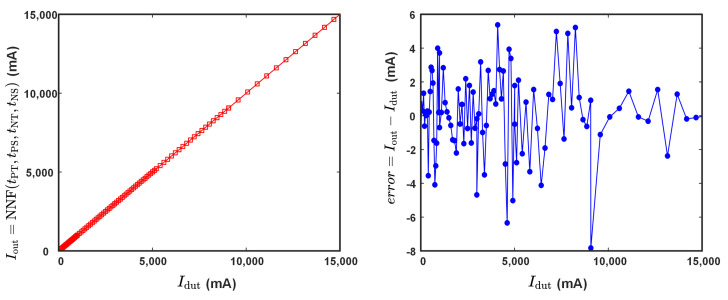
The sensor output characteristics utilizing neural networks. (**a**) The sensor calibration curve, (**b**) The sensor output error.

The trained neural network facilitates the evaluation of the sensor’s output characteristics, as shown in Figure 16. The maximum discrepancy between the sensor calibration curve and the actual values is approximately 7.83 mA, with a nonlinear error of around 0.054%. Compared to the results from the traditional RTD approximation presented in Figure 14, the newly designed sensor reduces the linearity error by more than 40 times and increases the effective measurement range by 150%. The detailed performance comparison is shown in Table 2. This demonstrates that the neural network approach significantly improves the sensor’s linearity and reduces the impact of ambient magnetic field interference.

A comparison of comprehensive performance parameters between our sensor and state-of-the-art fluxgate current sensors with similar measurement ranges is presented in Table 3. The comparison indicates that our developed sensor exhibits significant advantages in terms of linearity error and accuracy.

## 6. Discussion

This paper introduces a novel fluxgate current sensor that utilizes the magnetization residence times effect and neural networks. The M wave is found in the fluxgate current sensor for the first time, enabling simultaneous detection of ambient magnetic fields and current-induced annular magnetic fields. Neural networks are utilized to mitigate magnetic disturbances based on the aforementioned phenomenon. The performance enhancement method was verified through theoretical analysis and laboratory experiments. Finally, a current sensor with a linearity error of 0.054% within a 15 A measurement range was fabricated.

The sensor introduced in this study operates primarily in the time domain. With the proposed signal conditioning circuits, the sensor can achieve a digital output without the need for analog-to-digital conversion, making it compatible with low-cost controllers equipped with counters or timers. The incorporation of neural networks into our design has brought significant improvements to the sensor. Compared to the traditional RTD approximation method, neural networks effectively extend the measurement range, improve linearity, and suppress magnetic interference. Integrating more artificial intelligence methods into traditional physical sensors can lead to substantial advancements, especially in applications requiring a balance between cost and performance. Currently, we utilize static neural networks in fluxgate current sensors, which do not qualify as active intelligent sensors. In our future work, we aim to integrate FPGAs into our sensors and embed dynamic neural networks to implement real-time training, enabling the sensors to have active control capabilities.

## Figures and Tables

**Table 1:** The parameters of cobalt-based amorphous alloy film.

Thickness	Composition	Saturation Density	Magnetic Coercivity	Maximum Permeability
20 µm	${\mathrm{Co}}_{65}$ ${\mathrm{Fe}}_{5}$ ${\mathrm{Cr}}_{2}$ ${\mathrm{Si}}_{18}$ $B_{10}$	0.48 T	0.22 A/m	>580,000

**Table 2:** Performance comparison between sensors using neural network-enhanced RTD and traditional RTD.

Model	Measurement Range (A)	Linearity Error (%)
Sensor with nerual network combined RTD	15	0.054
Sensor with the traditional RTD	6	2.17

**Table 3:** Comparison of performance parameters.

Model	Measurement Range (A)	Linearity Error (%)	Accuracy (mA)
The designed sensor	15	0.054	7.83
HF-A06V0625PP5D [45]	18	0.1	18
Sensors in [46]	20	0.25	50

## Data Availability

The data presented in this study are available upon request from the authors.
